# Financial well-being: Capturing an elusive construct with an optimized measure

**DOI:** 10.3389/fpsyg.2022.935284

**Published:** 2022-08-12

**Authors:** Marc Aubrey, Alexandre J. S. Morin, Claude Fernet, Noémie Carbonneau

**Affiliations:** ^1^Department of Psychology, Université du Québec à Trois-Rivières, Trois-Rivières, QC, Canada; ^2^Department of Psychology, Concordia University, Montréal, QC, Canada; ^3^Department of Human Resources Management, Université du Québec à Trois-Rivières, Trois-Rivières, QC, Canada

**Keywords:** financial well-being, scale validation, Multidimensional Subjective Financial Well-being Scale, bifactor, exploratory structural equation modeling

## Abstract

Several definitions and measures of financial well-being (FWB) have been proposed in the scientific literature. The Multidimensional Subjective Financial Well-being Scale (MSFWBS) stands out among these measures in its ability to account for the conceptual richness of FWB. However, the original validation study based on a confirmatory factor analytic model indicated that the factor structure of scores obtained on this instrument was acceptable at best, revealing factor correlations high enough to question the discriminant validity of the factors. To improve conceptual and operational clarity of FWB, this study assesses the psychometric properties of the MSFWBS among French-Canadian adults (*n* = 454), using statistical models better suited to the examination of multidimensional constructs (exploratory structural equation modeling—ESEM, and bifactor-ESEM). Our results supported a bifactor-ESEM representation of scores on the MSFWBS, and their measurement invariance across groups of participants defined on the basis of their age, sex, personal income and household income. Our results also supported the convergent (with other measures of FWB) and criterion-related (with measures of life satisfaction, perceived stress, and psychological distress) validity of scores obtained on the MSFWBS (particularly the global FWB factor). By providing an optimized measure of FWB, our study contributes to advancing research on FWB.

## Introduction

“*Do not be fooled, financial well-being can be measured*” (Tison, 2019).

This quote, taken from a newspaper article, refers to a study carried out by the Financial Consumer Agency of Canada in 2019 to assess financial well-being (FWB). Journalists are not the only ones interested in FWB. Given the role attributed to FWB as a key predictor of employees’ performance and health, this construct has been heavily marketed by international organizations (e.g., OECD/INFE, 2020), financial institutions (e.g., Desjardins, 2020; Santander, 2020; HSBC, 2021) and insurance companies (e.g., SSQ, 2016; MetLife, 2019; Sun Life Financial, 2020). Recognizing the importance of FWB, numerous employers are now offering financial wellness programs to their employees (Bank of America, 2021). Excellence Canada (2019) even issues certifications to organizations implementing best practices for supporting employees’ well-being, including one specific to FWB. Despite this growing interest, the scientific community has been slow to follow suit, with research only recently beginning to address FWB (Nanda and Banerjee, 2021; Vörös et al., 2021).

Although FWB is likely to positively influence physical and psychological well-being (Shim et al., 2009; Netemeyer et al., 2018), efforts to properly operationalize this construct remain limited. For instance, although most operationalizations position FWB as a multidimensional construct, they do so based on inconsistent definitions in which the nature and number of dimensions vary greatly. This study seeks to fill this gap by refining and broadening our understanding of the multidimensional nature of FWB. To this end, we start by critically examining and comparing existing measures of FWB to identify the core dimensions essential to a comprehensive operationalization of FWB. Of those measures, one stood out in its ability to provide a comprehensive coverage of FWB: the Multidimensional Subjective Financial Well-Being Scale (MSFWBS; Sorgente and Lanz, 2019). We thus proceeded to a more rigorous assessment of the psychometric properties of scores obtained on this measure. Our conceptual analysis and empirical examination of this promising measure help to increase our understanding of FWB, while proposing a foundation upon which to build further theoretical and empirical developments with practical implications.

## From general well-being to financial well-being

Unlike studies on general well-being, research on FWB is still fragmented and incomplete. This fragmentation can be partly attributed to the fact that the scientific community has not yet reached consensus on how to define FWB (Brüggen et al., 2017), leading to multiple inconsistencies in its operationalization (Sorgente and Lanz, 2017). More alarming is the fact that many studies have been carried out despite the lack of a clear conceptual definition of FWB (Brüggen et al., 2017). To address this issue, we first turn our attention to the definition of the broader general well-being construct, before addressing the operational definition of the more specific FWB construct.

### What is general well-being?

General well-being is usually studied from two distinct perspectives: subjective well-being (SWB; e.g., life satisfaction, positive and negative affect, etc.) and psychological well-being (PWB; e.g., self-actualization, development, etc.; Keyes et al., 2002). SWB encompasses an affective and a cognitive component (Diener et al., 2017). The affective component refers to individuals’ experience of pleasurable emotions not tainted by negative emotions, whereas the cognitive component refers to the extent to which they feel satisfied with specific areas of their life (e.g., employment, health) or with their life in general (Diener et al., 2017). SWB thus involves a hedonic view of well-being, centered on life enjoyment. In contrast, PWB entails an eudaimonic view of well-being, centered on positive functioning and fulfillment (Keyes et al., 2002). Ryff (1989) defined PWB as encompassing six components: (1) self-acceptance (i.e., positive regard toward oneself, acceptance of positive and negative aspects of self), (2) positive relations with others (i.e., warm and trusting relationships with others), (3) autonomy (i.e., a sense of independence and self-determination), (4) environmental mastery (i.e., sense of competence and discipline in managing one’s environment), (5) life purpose (i.e., having oriented goals and objectives), and (6) personal growth (i.e., self-development and self-actualization). Research has generally shown that SWB and PWB represent distinct, but interrelated, constructs that together provide a comprehensive overview of general well-being (Keyes et al., 2002; Chen et al., 2013; Disabato et al., 2016). Keyes et al. (2002) proposed the label *optimized* well-being to describe the joint experience of SWB and PWB.

### What is financial well-being?

Although FWB has been operationalized in multiple manners, six operationalizations have stood out in research. The definition of FWB used in these six main operationalizations, as well as a comparison of their core components, are presented in Table 1. Examining this Table reveals four common characteristics. First, all of these definitions highlight the multidimensional nature of FWB. Second, all definitions note that FWB entails a perception of having access to an income seen as adequate to meet one’s needs. Third, all definitions entail a temporal perspective focused on one’s future FWB expectations. Fourth, all definitions emphasize the idea that FWB involves a cognitive evaluation of one’s financial situation as satisfactory. Beyond these common characteristics, some other components are less consensual. Thus, only four, out of six, definitions refer to FWB as a state characterized by the perceived ability to enjoy life, or as a state involving an otherwise positive emotional experience. Likewise, only three definitions highlight the importance of being in control of one’s financial situation. In these definitions, this sense of control is intimately related to the emotional component of FWB, which is consistent with the well-documented role of control in stress perceptions (e.g., Averill, 1973; Lazarus and Folkman, 1984; Sherman and Mehta, 2020). However, despite this connection, this sense of control remains distinct from this emotional component and involves individuals’ ability to plan and manage their finances on their own. Finally, only Sorgente and Lanz (2019) incorporated a social comparison component to their operationalization, according to which FWB is seen as depending in part from individuals’ assessment of their financial situation in a positive light relative to that of others. Indeed, these authors note that peer comparisons seem particularly relevant for emerging adults (aged 18–29), which were the focus of their work and might explain why others did not consider this facet of FWB. Others also noted the relevance of social comparison when referring to FWB (e.g., Porter and Garman, 1993; Brown and Gray, 2016), thus highlighting the broad relevance of this dimension for FWB measurement.

**Table 1:** Definitions and dimensions of financial well-being.

**Author (s)**	**Definition**						
Consumer Financial Protection Bureau (CFPB, 2015)	A state of being wherein individuals can fully meet their current and ongoing financial obligations, can feel secure in their financial future, and be able to make choices that allows them to enjoy life.						
Kempson and Poppe (2017)	The extent to which individuals can meet all of their current commitments and needs comfortably and have the financial resilience to maintain this ability in the future.						
Muir et al. (2017)	Financial wellbeing occurs when individuals are able to meet their expenses with some money left over, are in control of their finances, and feel financially secure now and in the future.						
Brüggen et al. (2017)	Individuals’ perceptions of being able to sustain current and anticipated desired living standards and financial freedom.						
Netemeyer et al. (2018)	No original definition was proposed by the authors, who relied on definitions provided by others.						
Sorgente and Lanz (2017, 2019)	Subjective financial well-being corresponds to individuals’ emotional and cognitive evaluation of their own financial condition, that is to their subjective experiences of that condition.						
**Dimensions**							
Author (s)	**Income adequacy**	**Life Enjoyment**	**Temporal (present vs. future)**	**Relativity (vs. others)**	**Control (financial management)**	**Cognitive evaluation (satisfaction)**	**Emotional evaluation (stress, anxiety)**
CFPB (2015)	X	X	X			X	X
Kempson and Poppe (2017)	X	X	X			X	
Muir et al. (2017)	X	X	X		X	X	X
Brüggen et al. (2017)	X	X	X			X	
Netemeyer et al. (2018)	X		X		X	X	X
Sorgente and Lanz (2017, 2019)	X		X	X	X	X	X

None of these operationalizations considered individuals’ objective financial situation (e.g., income level, net worth, etc.) as the sole, or even as a core, component of FWB. Rather, they all emphasized the multidimensional and subjective nature of FWB as a state encompassing cognitive and affective components. This recognition suggests a conceptual similarity between FWB and the more specific construct of financial satisfaction (Sorgente and Lanz, 2017), reflecting individuals’ cognitive assessment of their financial situation as satisfactory (e.g., Joo and Grable, 2004; Plagnol, 2011; Brown et al., 2014; Brzozowski and Spotton Visano, 2019). By incorporating an affective and temporal perspective, these definitions thus position FWB as more generic than financial satisfaction.

Based on this synthesis, we define FWB as a positive psychological state characterized by a sense of contentment about one’s personal financial situation and by a positive perception of one’s financial situation as able to actively meet one’s current needs and future aspirations. This operational definition is aligned with the typical definition of general well-being as encompassing a hedonic and an eudaimonic component (Keyes et al., 2002), while being specific to one’s financial situation.

### How is financial well-being assessed?

With the ambiguity surrounding the operationalization of FWB, it is not surprising that FWB has been measured in many different ways across studies, making it hard to compare results. Based on the type of measure used, studies can be grouped into four categories, based on whether they relied on: (1) an in-house single-item measure (e.g., Brown and Gray, 2016; Lui et al., 2016); (2) an in-house measure for which psychometric properties remain unknown (e.g., Momentum and UNISA, 2011; Serido and Shim, 2017; Burcher et al., 2021); (3) a measure for which information on psychometric properties is provided (e.g., factor structure, construct validity; Prawitz et al., 2006; CFPB, 2017; Kempson and Poppe, 2017; Netemeyer et al., 2018; Sorgente and Lanz, 2019); (4) a measure adapted from those included in the previous category, and thus not directly relevant in their own right (e.g., Ponchio et al., 2019; Brenner et al., 2020; Utkarsh et al., 2020).

Among the measures forming the third category (for which psychometric properties are reported), two rely on a one-dimensional structure of FWB (Prawitz et al., 2006; CFPB, 2017), which represents a major shortcoming given the emerging consensus outlined in the previous section regarding the multidimensional nature of FWB. Moreover, albeit relying on measures specifically designed to reflect multiple components of FWB, CFPB (2017), Kempson and Poppe (2017), and Prawitz et al. (2006) all failed to find support for the multidimensional nature of these measures, rather providing evidence for a unidimensional structure. In contrast, Netemeyer et al.’s (2018) Perceived Financial Well-Being Scale (PFWBS) and Sorgente and Lanz’s (2019) MSFWBS were both found to follow a multidimensional structure. However, Netemeyer et al.’s (2018) PFWBS only focuses on two dimensions of FWB (i.e., current money management stress and expectations in terms of future financial security). In contrast, Sorgente and Lanz’s (2019) MSFWBS provides a way to differentiate between five components of FWB *via* a total of 25 items: (1) General Subjective Financial Well-Being (GS); (2) Money Management (MM); (3) Peer Comparison (PC); (4) Having Money (HM) and (5) Financial Future (FF).

As a result, the MSFWBS not only differentiates between respondents’ current (GS, MM, PC, HM) and future (FF) perspectives, it also incorporates affective and cognitive components of FWB, while being the only one to consider social comparisons (PC). Through its ability to differentiate between cognitive and affective components of FWB, the MSFWBS thus shares a natural connection with generic measures of well-being (affective FWB: Hedonic SWB; cognitive FWB: Eudaimonic PWB), consistent with our positioning of FWB as a component of general well-being. In fact, the MSFWBS is related to the only operationalization included in Table 1 that covers all of the key elements proposed to be associated with FWB, with a single exception (i.e., life enjoyment). Indeed, unlike other conceptualizations of FWB as well as Keyes et al.’s (2002) representation of optimized well-being, Sorgente and Lanz (2019) did not consider the financial ability to make choices allowing one to enjoy life as a distinct component of FWB. However, despite not being reflected as a specific dimension of FWB, enjoyment is still considered in the MSFWBS in the form of indicators of the affective GS dimension (i.e., “I have enough money to pursue my passions” and “I have enough funds to enjoy my life”). For all of these reasons, the MSFWBS was retained for the present investigation.

### The Multidimensional Subjective Financial Well-being Scale

Despite its interest, validity evidence for the MSFWBS currently only comes from a sample of emerging adults recruited in Italy and Portugal (Sorgente and Lanz, 2019). As a result, the extent to which the psychometric properties of scores on this instrument would generalize to a wider age range and to different cultural or linguistic groups remains unknown. Considering the glass ceiling to which women still tend to be exposed in modern organizations (Stamarski and Son Hing, 2015), the widespread range of income discrepancies observed in the workplace, and the key influence of income on different measures of general well-being (Kahneman and Deaton, 2010), it appears critical to verify whether the measurement of FWB can be expected to generalize as a function of these characteristics.

Moreover, evidence for the factor validity of scores on the MSFWBS provided by Sorgente and Lanz (2019) was obtained through confirmatory factor analyses (CFA), which revealed factor correlations that were high enough to call into question the discriminant validity of some factors (i.e., *r* = 0.793–0.811 for the correlations between HM, PC, and GS). These correlations, however, are not surprising given that the MSFWBS is specifically designed to assess conceptually related facets of FWB. In this situation, statistical research has revealed that measurement models benefit from relying on exploratory structural equation modeling (ESEM) to achieve a more accurate representation of the structure of multidimensional instruments (Asparouhov and Muthén, 2009; Marsh et al., 2009; Alamer and Marsh, 2022; Shao et al., 2022). ESEM makes it possible to freely estimate all cross-loadings between items and non-target factors (Morin et al., 2016a, 2020). This less constrained estimation procedure makes it possible to assess factors using all of the relevant information included in all of the items, and has been demonstrated to result in more accurate estimates of the factors, their correlations, and their associations with other constructs, while remaining unbiased when cross-loadings prove to be unnecessary (Asparouhov et al., 2015; Mai et al., 2018; Alamer and Marsh, 2022; Shao et al., 2022). For instance, although the item “I have enough money to pursue my passions” is an indicator of the GS subscale, incorporating cross-loadings would make it possible to acknowledge that this item also incorporates a future perspective (FF) and the availability of enough money (HM) resulting from an efficient management of one’s assets (MM), although these additional associations are likely to be much smaller than the one involving the GS factor. Moreover, the estimation of ESEM measurement models using target rotation procedures (Browne, 2001; Asparouhov and Muthén, 2009) makes it possible to rely on an *a priori* specification of the main indicators of each factor while targeting all cross-loadings to remain as close to 0 as possible.

Moreover, although the name of the GS factor suggests that it might reflect the globality of FWB, this factor is rather conceptualized as reflecting a more hedonic form of subjective financial well-being. For instance, this factor is the only one who incorporates items related to life enjoyment, seen by some as critical to FWB (CFPB, 2015; Brüggen et al., 2017), as well as items reflecting the affective nature of FWB (“I am constantly stressed because of my financial situation” and “I am calm about my financial situation”). Thus, despite Sorgente and Lanz’s (2019) suggestion that this factor could be used as a single indicator of FWB in studies seeking a shorter measure, doing so would involve ignoring the eudaimonic components of FWB, as well as the future orientation component highlighted as critical in many previous operationalizations (see Table 1).

The distinctive nature of the GS factor does not mean that the MSFWBS is unable to provide evidence of respondents’ global levels of FWB. Indeed, this instrument is specifically designed to assess what are seen as different components of a single overarching construct. In this situation, previous research conducted on the construct of general well-being (e.g., Morin et al., 2016b, 2017) have demonstrated the value of relying on a bifactor-ESEM representation to adequately capture the inherent duality (global/specific) of this construct. More precisely, a bifactor model (which can be estimated with CFA or ESEM) would directly estimate participants’ global levels of FWB from the variance shared among all items used in the questionnaire, while also providing a non-redundant estimate of the specificity associated with each subscale (HM, PC, GS, MM, and FF) beyond the variance already explained by the G-factor (Morin et al., 2016a, 2020).

### The present study

This study aimed to conduct an in-depth examination of the psychometric properties of scores obtained on the MSFWBS. First, we examined the factor structure of these scores while contrasting CFA, bifactor-CFA, ESEM and bifactor-ESEM solutions. Second, we examined the measurement invariance of these scores across subsamples defined based on age, sex, personal income, and household income. Third, we examined the convergent validity of these scores in relation to other validated measures of FWB: (a) the PFWBS (Netemeyer et al., 2018); (b) the Financial Well-Being Scale (CFPB, 2017); and (c) the Financial Anxiety Scale (Shapiro and Burchell, 2012). We finally examined the criterion-related validity of these scores in relation to validated measures of theoretically related constructs (i.e., perceived stress, psychological distress and life satisfaction). Indeed, FWB has been shown to be related to general well-being (Brüggen et al., 2017; Muir et al., 2017; Netemeyer et al., 2018), life satisfaction (Shim et al., 2009; Brzozowski and Spotton Visano, 2019; Sorgente and Lanz, 2019), and psychological distress (Espinosa and Rudenstine, 2020).

## Materials and methods

### Procedure and participants

Participants were recruited from a medium-sized University located in the Canadian Province of Quebec and were recruited using direct contacts and Facebook ads. A total of 454 participants (*M_age_* = 34.9 years; 18–81 years) including 308 women (67.8%) and 146 men (32.2%), completed an online questionnaire in the Fall of 2019. As an incentive, participants were told that 1% of them would randomly receive a compensation of CAD$50 for their participation. A significant proportion (33.2%) of respondents were under the age of 25 years and 46% of them were students. Emerging adults (aged 18–29), the only age group studied by Sorgente and Lanz (2019), thus represented 45.8% of our sample. In terms of personal gross (pre-tax) income, 47.6% of the respondents earned <CAD$40,000 per year, and 55.6% had a gross household income of <CAD$90,000, while 30% had a gross household income >CAD$120,000. When compared to the 2019 Quebec population (Institut de la statistique du Québec, 2020), our sample included more women (67.8% vs. 50%), had a higher level of education (66% vs. 32.8% had a university degree), and was younger (*M_age_* = 34.9 vs. 42.4 years). Our income measure relied on interval scaling, which made it impossible to determine participants’ exact income. However, considering that 36.7% of the Quebec population reported a net (after-tax) household income over CAD$60,000, it seems reasonable to conclude that the income of our sample is slightly higher given that 45% of our respondents reported a gross (before tax) personal income higher than CAD$90,000. All participants voluntarily provided informed consent for their participation and were ensured of the confidentiality of their responses. The study protocol was approved by the research ethics committee of the targeted University.

### Measures

Measures were adapted to French using a standardized translation back translation procedure involving bilingual members of the research team (Hambleton, 2005). Model based composite reliability (*ω*) is reported later for all instruments when we describe their final measurement models.

#### Financial well-being

Participants rated the 25 items (reported in Appendix) from the MSFWBS. They also completed the two five-item subscales from the PFWBS (future expectations, *α* = 0.872; e.g., “I am securing my financial future”; current situation, *α* = 0.840; e.g., “My finances control my life”), the 10 items from the CFPB’s financial well-being scale (*α* = 0.890; e.g., “I could handle a major unexpected expense”) and eight items (out of 10) of the FAS (*α* = 0.884; e.g., “I prefer not to think about the state of my personal finances”). Although the original FAS includes 10 items, two of them were excluded in our study as they were only relevant to student populations (“I do not think I am doing as well as I could academically because I worry about money” and “I am worried about the debt I will have when I complete my university education”). All items were rated on a five-point scale (1-absolutely false to 5-absolutely true). Importantly, four items are common to the PFWBS and CFPB. We describe in the analysis section how this specificity was handled in the analyses.

#### Criterion measures

Participants rated the 14 items (e.g., “In the last month, how often have you felt that you were on top of things?”) from the Perceived Stress Scale (Cohen et al., 1983; *α* = 0.871) using a five-point scale ranging from (0) Never to (4) Very often. They also rated the six items (e.g., “During the last 30 days, about how often did you feel so depressed that nothing could cheer you up?”) from Kessler et al.’s (2002) Psychological Distress Scale (K6; *α* = 0.873) using a five-point scale ranging from (1) All of the time to (5) None of the time. Finally, they rated the five items (e.g., “I am satisfied with my life”) from the Satisfaction with Life Scale (Diener et al., 1985; *α* = 0.904) using a seven-point scale ranging from (1) Strongly disagree to (7) Strongly agree.

## Analyses

### Model estimation

All analyses were conducted using the Mplus 8.4 statistical package and the maximum likelihood robust estimator (MLR), which is robust to non-normality (Rhemtulla et al., 2012). Due to the way our online questionnaire was programmed, there were no missing responses. The degree to which each model was able to provide an adequate approximation of the data was assessed using several statistical indicators: The chi-square test of exact fit (*χ*^2^), the comparative fit index (CFI), the Tucker-Lewis index (TLI), as well as the root mean square error of approximation (RMSEA) and its 90% confidence interval. Given the known oversensitivity of the *χ*^2^ to sample size, minor misspecifications, and even omitted variables (e.g., Marsh et al., 2005) this indicator will be reported, but not interpreted. Values >0.90 for the CFI and TLI are considered acceptable, but these values should ideally be >0.95 (Hu and Bentler, 1999; Marsh et al., 2005). Likewise, RMSEA values lower than 0.08 are acceptable but should ideally be lower than 0.06. (Hu and Bentler, 1999; Marsh et al., 2005).

### Main measurement models

The structure of participants’ responses to the MSFWBS was investigated by comparing CFA, bifactor-CFA, ESEM, and bifactor-ESEM solutions. In the CFA solution, MSFWBS responses were assumed to reflect five correlated factors (HM, PC, GS, MM, and FF), each factor was only defined by its *a priori* indicators, and no cross-loadings were allowed. The ESEM solution involved the assessment of the same five factors. However, all cross-loadings were freely estimated but targeted to take a value as close to zero as possible *via* an oblique target rotation procedure (Browne, 2001). In the bifactor-CFA solution, MSFWBS responses to all items were allowed to define a global FWB factor (G-factor), in addition to their *a priori* factors (S-factors; HM, PC, GS, MM, and FF). As in CFA, no cross-loadings were allowed in this model. Finally, the bifactor-ESEM solution, the G-and S-factors were specified as in the bifactor-CFA solution, but all cross-loadings were freely estimated between the S-factors, although they were again targeted to take a value as close to zero as possible *via* an orthogonal bifactor target rotation procedure (Reise et al., 2011). According to typical bifactor assumptions, both bifactor solutions were specified as orthogonal (the factors were uncorrelated; Morin et al., 2020). One orthogonal method factor was added to all models to control for the methodological artifact related to the negative wording of seven of the items (marked in italics in Appendix; Marsh et al., 2010).

The comparison of these four models was done following a sequential strategy outlined by Morin et al. (2016a,b, 2020). The CFA and ESEM solutions were first contrasted to assess the relevance of incorporating cross-loadings. Observing that the ESEM solution is able to achieve a higher level of fit to the data, that the factor correlations are reduced in the ESEM solution relative to its CFA counterpart, that all factors remain equally well-defined in both solutions, and that ESEM cross-loadings remain either small or easily explainable can all be taken as evidence favoring the ESEM solution. The observation of multiple non-negligible cross-loadings in ESEM may also suggest the presence of an unmodeled G-factor (i.e., the need for a bifactor representation). The retained solution (CFA or ESEM) was then contrasted with its bifactor counterpart. Observing a higher level of fit associated with the bifactor solution, the presence of a well-defined G-factor coupled with at least a subset of well-defined S-factors, and reduced cross-loadings relative to the ESEM solution can all be taken as evidence supporting the bifactor solution.

### Measurement invariance

The measurement invariance of the retained solution was tested according to the following sequence (Millsap, 2011): *configural* (same model with no additional constraint), *weak* (equal factor loadings), *strong* (equal factor loadings and item intercepts), *strict* (equal factor loadings, item intercepts and item uniquenesses), latent variance–covariance (equal factor loadings, item intercepts, item uniquenesses, and latent variances and covariances), and latent mean invariance (equal factor loadings, item intercepts, item uniquenesses, latent variances and covariances, and latent means). These tests of measurement invariance were conducted across subgroups of participants defined on the basis of: (a) sex [males (*n* = 146) vs. females (*n* = 308)]; (b) age [emerging adults aged 18–29 (*n* = 203) vs. adults aged over 30 (*n* = 240)]; (c) personal income [CAD$40,000 or less (*n* = 215) vs. more than CAD$40,000 (*n* = 235)]; (d) household income [CAD$90,000 or less (*n* = 250) vs. more than CAD$90,000 (*n* = 200)]. Model comparisons relied on an examination of changes in CFI, TLI and RMSEA between each model and the previous one. A decrease in the value of the CFI or TLI lower or equal to 0.010 or an increase in the value of the RMSEA higher or equal to 0.015 for the RMSEA was considered to support the invariance (Cheung and Rensvold, 2002; Marsh et al., 2005; Chen, 2007).

### Convergent validity

To assess convergent validity of scores obtained on the MSFWBS, we estimated latent correlations between the MSFWBS factors (from the optimal model retained previously) and latent factors representing the other measures of FWB (PFWBS, CFPB, FAS). In this model, the four items used in both the PFWBS and CFPB were removed from the CFPB, the PFWBS was modeled according to a bifactor-ESEM operationalization matching that retained for the MSFWBS, and the CFPB (*ω* = 0.861 for the reduced version and 0.894 for the full version) and FAS (*ω* = 0.896) were each modeled as a single factor. Like in the main model, an orthogonal method factor was used to account for the negative wording of 23 items (7 MSFWBS items, 3 items from the shortened CFPB, 5 PFWBS items, and all 8 FAS items). The parameter estimates from the measurement component of this model, in relation to the convergent measures, are reported in Supplementary Table S3 of the online supplements. These results support the proper definition of all factors, while also indicating that the PFWBS items related to future expectations mainly serve to define the global FWB, leaving little specificity associated with the future expectations S-factor. To assess the convergent validity of the MSFWBS factors in relation to the complete CFPB, a second model was estimated including only the MSFWBS factors and the CFPB (a single factor including all items). This model also included an orthogonal method factor related to the 13 negatively worded items (7 MSFWBS items and 6 CFPB items).

### Criterion-related validity

The criterion-related validity of scores obtained on the MSFWBS was assessed by allowing these scores to predict scores on three latent CFA factors representing the outcome variables (perceived stress: *ω* = 0.887; psychological distress: *ω* = 0.904; life satisfaction: *ω* = 0.914) in a fully latent predictive model. The negative wording of the seven MSFWBS items and of seven items from the Perceived Stress Scale was controlled as in the in the previous stages of the analyses. The parameter estimates from the measurement part of this model, in relation to the criterion measures, are reported in Supplementary Table S4 in the online supplements and support the proper definition of all factors.

## Results

### Factor structure of scores obtained on the MSFWBS

The model fit associated with the alternative MSFWBS measurement models are reported in Table 2, and parameter estimates from these models are reported in Table 3 (CFA and ESEM factor correlations), 4 (factor loadings and item uniquenesses), and 5 (omega coefficients of composite reliability). All models achieved an acceptable (CFA, Bifactor-CFA, ESEM) to excellent (Bifactor-ESEM) level of fit to the data. Following the sequential strategy outlined by Morin et al. (2016a,b, 2020), we first considered the CFA and ESEM solutions. Although both solutions resulted in an acceptable level of fit, the fit of the ESEM solution was substantially higher than that of the CFA solution (ΔCFI = +0.039, TLI = +0.023, RMSEA = −0.008). Moreover, the factor correlations were substantially reduced in ESEM (*r* = 0.345–0.696; *M_r_* = 0.534) relative to CFA (*r* = 0.669–0.903; *M_r_* = 0.795). Finally, with few exceptions associated with the ESEM solution, both the CFA (*|λ|* = 0.570–0.918; *M_|λ|_* = 0.790) and ESEM (*|λ|* = 0.069–0.993; *M_|λ|_* = 0.561) solutions resulted in factors that were reasonably well-defined and reliable (CFA: *ω* = 0.860–0.944; ESEM: *ω* = 0.758–0.901).

**Table 2:** Fit of the alternative measurement models estimated for the MSFWBS.

	*χ* ^2^	df	CFI	TLI	RMSEA	RMSEA 90% CI
CFA	790.548^*^	258	0.917	0.903	0.067	0.062; 0.073
Bifactor-CFA	679.328^*^	243	0.932	0.916	0.063	0.057; 0.069
ESEM	460.046^*^	178	0.956	0.926	0.059	0.052; 0.066
Bifactor-ESEM	327.082^*^	158	0.974	0.950	0.049	0.041; 0.056

*χ*^2^. robust chi-square test of exact fit; df, degrees of freedom; CFI, comparative fit index; TLI, Tucker-Lewis index; RMSEA, root mean square error of approximation; 90% CI, 90% confidence interval; CFA, Confirmatory factor analysis; ESEM, Exploratory structural equation modeling.

* *p* < 0.01.

**Table 3:** Factor correlations for the CFA and ESEM measurement models estimated for the MSFWBS.

	CFA				ESEM			
	HM	PC	GS	MM	HM	PC	GS	MM
PC	−0.801^**^				−0.487^**^			
GS	−0.806^**^	0.856^**^			−0.612^*^	0.696^**^		
MM	−0.669^**^	0.720^**^	0.879^**^		−0.345^**^	0.347	0.579	
FF	−0.675^**^	0.772^**^	0.871^**^	0.903^**^	−0.534^**^	0.498	0.691^**^	0.546^*^

CFA, Confirmatory factor analysis; ESEM, Exploratory structural equation modeling; HM, Having money; PC, Peer Comparison; GS, General subjective financial well-being; MM, Money Management; FF, Financial future.

* *p* ≤ 0.05 and

** *p* ≤ 0.01.

Moreover, the ESEM solution evidenced multiple statistically significant cross-loadings (*|λ|* = 0.003–0.544; *M_|λ|_* = 0.120), thus supporting the need to account for this form of multidimensionality (Asparouhov et al., 2015), and even suggesting the possible presence of an unmodeled G-factor (Morin et al., 2020). Some cross-loadings, however, were higher than expected. For instance, item HM2 presented cross-loadings (−0.282, −0.489, and − 0.287) higher than its main loading (0.111) on three other factors, suggesting that this item might be a better indicator of global levels of FWB than of any specific factor. Similarly, items GS1, MM2, and FF5 had a substantial cross-loading (respectively, 0.399, 0.378, and 0.371) on one secondary factor, even though these cross-loadings were of a similar magnitude, or slightly lower, than their main loading (respectively, 0.372, 0.464, and 0.490). Once again these cross-loadings suggest that these items might contribute to the definition of global levels of FWB more than to the definition of their own factors. More problematic were items GS7 and FF4, presenting a cross-loading (respectively, −0.479 and 0.544) higher than their main loading (respectively, 0.069 and 0.203), suggesting that these items might have been assigned to the wrong factor. Item GS7 (*I have enough funds for everything I need*) corresponded more closely to the HM factor than to the GS factor, whereas FF4 [*I am satisfied with the way I am preparing myself to reach my long-term goals (for example, to buy a car)*] correspond to the MM factor more than to the FF factor. Interestingly, this interpretation is well-aligned with the content of these items, suggesting that our results might be more aligned with the proper assignment of these items to, respectively, factors HM and MM (Tables 4, 5).

**Table 4:** Standardized parameter estimates from the alternative measurement models estimated for the MSFWBS.

Item	CFA		Bifactor CFA			ESEM						Bifactor ESEM						
	*λ*	*δ*	G-*λ*	S-*λ*	*δ*	HM *λ*	PC *λ*	GS *λ*	MM *λ*	FF *λ*	*δ*	G-*λ*	HM *λ*	PC *λ*	GS *λ*	MM *λ*	FF *λ*	*δ*
HM1	0.858	0.256	−0.623	*0.429*	0.230	**0.609**	*−0.047*	*−0.176*	*0.027*	*−0.075*	0.230	**−0.670**	**0.428**	*−0.001*	*0.020*	*0.073*	*−0.019*	0.228
HM2	0.720	0.299	0.59	*0.076*	0.287	***0.111***	*−0.282*	*−0.489*	*0.137*	*−0.007*	0.287	**−0.692**	***0.024***	*0.041*	*0.119*	0.185	*0.080*	0.276
HM3	0.869	0.243	−0.649	*0.602*	0.184	**0.763**	*−0.047*	*−0.062*	*−0.093*	*0.008*	0.184	**−0.678**	**0.537**	*0.030*	*−0.013*	*−0.006*	*0.022*	0.184
PC1	0.866	0.250	0.744	−0.582	0.285	*−0.177*	***0.599***	*−0.076*	*0.171*	*0.181*	0.285	**0.757**	*−0.065*	**−0.385**	*−0.066*	*0.035*	*0.044*	0.268
PC2	−0.748	0.412	−0.610	0.299	0.419	*0.195*	***−0.497***	*−0.037*	*−0.015*	*−0.114*	0.419	**−0.643**	*0.086*	**0.345**	*0.003*	0.090	*−0.009*	0.385
PC3	−0.713	0.137	−0.577	−0.29	0.202	*−0.057*	**−0.665**	*−0.257*	*0.095*	*−0.013*	0.202	**−0.675**	*−0.110*	**0.298**	0.171	0.193	0.102	0.216
GS1	0.776	0.398	0.706	0.337	0.334	*−0.057*	0.399	***0.372***	*0.049*	*0.066*	0.334	**0.807**	*0.030*	*−0.078*	***−0.223***	*−0.038*	*−0.053*	0.288
GS2	−0.703	0.477	−0.645	−0.208	0.442	0.200	*−0.166*	**−0.460**	*−0.022*	*0.028*	0.442	**−0.706**	*0.087*	*0.044*	***−0.016***	*0.061*	*0.079*	0.443
GS3	0.800	0.360	0.732	0.331	0.351	−0.165	*0.140*	**0.519**	*0.065*	*0.040*	0.351	**0.803**	*−0.055*	*0.002*	***−0.005***	*−0.022*	*−0.040*	0.350
GS4	0.849	0.279	0.796	0.306	0.257	*0.082*	*0.161*	**0.602**	*0.103*	*0.166*	0.257	**0.850**	0.133	*−0.004*	***0.022***	*0.014*	*0.034*	0.258
GS5	−0.663	0.556	−0.622	*−0.157*	0.431	*0.141*	*0.252*	***−0.765***	*−0.062*	*0.044*	0.431	**−0.646**	*0.047*	*−0.156*	***−0.286***	*0.013*	*0.062*	0.405
GS6	0.825	0.319	0.823	*0.146*	0.283	*0.003*	*−0.077*	***0.606***	*0.243*	*0.164*	0.283	**0.805**	*0.072*	*0.034*	***0.269***	0.127	*0.065*	0.253
GS7	0.666	0.557	0.648	*0.127*	0.440	−0.479	*0.128*	***0.069***	*0.042*	0.183	0.440	**0.660**	−0.327	*−0.095*	***0.034***	*−0.023*	0.092	0.438
GS8	0.888	0.211	0.834	0.303	0.218	*−0.088*	*0.113*	**0.593**	*0.149*	*0.077*	0.218	**0.877**	*0.012*	*−0.022*	***0.103***	*0.032*	*−0.015*	0.218
GS9	0.869	0.244	0.816	0.308	0.202	*0.003*	*−0.075*	***0.834***	*0.091*	*0.076*	0.202	**0.866**	*0.079*	*0.093*	***0.215***	*−0.012*	*−0.014*	0.188
GS10	0.833	0.306	0.759	0.369	0.280	*−0.062*	*0.173*	**0.604**	*0.013*	*0.092*	0.280	**0.851**	*0.022*	*0.031*	***−0.053***	*−0.070*	*−0.019*	0.266
MM1	0.863	0.256	0.778	−0.48	0.234	*−0.005*	*0.080*	*0.171*	**0.682**	*0.062*	0.234	**0.727**	0.068	*0.063*	*−0.047*	**0.535**	*0.013*	0.174
MM2	0.809	0.346	0.746	0.332	0.353	*−0.041*	*0.030*	*0.378*	**0.464**	*0.022*	0.353	**0.748**	*0.035*	*0.140*	*−0.071*	**0.339**	*−0.029*	0.298
MM3	0.840	0.295	0.844	*0.092*	0.262	*−0.179*	*−0.023*	*0.148*	**0.439**	0.299	0.262	**0.757**	*−0.089*	*−0.030*	0.193	**0.307**	0.187	0.251
MM4	0.918	0.157	0.851	0.311	0.170	*−0.035*	*0.038*	*0.234*	**0.618**	0.139	0.170	**0.788**	*0.038*	*0.035*	*0.072*	**0.438**	*0.072*	0.174
FF1	0.748	0.440	0.694	0.309	0.391	*0.061*	0.176	*−0.018*	*0.083*	**0.676**	0.391	**0.651**	*0.056*	*−0.098*	*−0.016*	*0.077*	**0.406**	0.392
FF2	0.641	0.589	0.583	0.660	0.286	*0.044*	*−0.106*	*−0.044*	*−0.107*	**0.993**	0.286	**0.543**	*−0.007*	*0.077*	*−0.010*	*0.004*	**0.645**	0.282
FF3	0.570	0.676	0.529	0.512	0.427	*−0.024*	*−0.078*	*0.138*	−0.252	**0.797**	0.427	**0.550**	*−0.046*	*0.120*	*−0.087*	−0.134	**0.507**	0.398
FF4	0.850	0.278	0.853	*−0.077*	0.262	*−0.052*	*0.165*	*0.089*	0.544	**0.203**	0.262	**0.750**	*0.027*	*−0.153*	*0.170*	0.362	***0.110***	0.242
FF5	0.876	0.233	0.830	0.133	0.279	*−0.018*	*0.105*	*0.030*	0.371	**0.490**	0.279	**0.733**	*0.024*	*−0.126*	*0.157*	0.262	**0.305**	0.260

*λ*, Standardized factor loading; *δ*, Standardized item uniquenesses; G-, Global factor from a bifactor measurement model; S-, Specific factors from a bifactor measurement model; CFA, Confirmatory factor analysis; ESEM, Exploratory structural equation modeling; HM, Having money; PC, Peer Comparison; GS, General subjective financial well-being; MM, Money Management; FF, Financial Future; Item labels are reported in Appendix; Main ESEM (target) factor loadings are bolded; Non statistically significant parameters are marked in italics (*p* > 0.05).

**Table 5:** Composite reliability (*ω*) estimates from the alternative measurement models estimated for the MSFWBS.

	CFA	Bifactor-CFA	ESEM	Bifactor-ESEM	Final Bifactor-ESEM Solution
G-Factor		0.977		0.979	0.979
HM	0.882	0.636	0.758	0.587	0.620
PC	0.871	0.602	0.774	0.549	0.554
GS	0.944	0.675	0.901	0.326	0.388
MM	0.918	0.592	0.826	0.745	0.788
FF	0.860	0.635	0.858	0.712	0.720

CFA, Confirmatory factor analysis; ESEM, Exploratory structural equation modeling; HM, Having money; PC, Peer Comparison; GS, General subjective financial well-being; MM, Money Management; FF, Financial Future; G-factor, Global factor from a bifactor measurement model.

These results all support the value of an ESEM solution, which was retained for comparison with the bifactor-ESEM solution. This solution resulted in an excellent level of fit to the data, and in a much higher level of fit than the ESEM solution (ΔCFI = +0.018, TLI = +0.024, RMSEA = −0.010). It also resulted in the estimation of a very well-defined (*|λ|* = 0.543–0.877; *M_|λ|_* = 0.729) and reliable (*ω* = 0.979) G-factor, consistent with the idea that all items contribute to the definition of global levels of FWB. This solution also resulted in S-factors representing participants’ specific levels of items HM (*|λ|* = 0.024–0.537; *M_|λ|_* = 0.330; *ω* = 0.587), PC (*|λ|* = 0.298–0.385; *M_|λ|_* = 0.343; *ω* = 0.549), MM (*|λ|* = 0.307–0.535; *M_|λ|_* = 0.405; *ω* = 0.745), and FF (*|λ|* = 0.110–0.645; *M_|λ|_* = 0.395; *ω* = 0.712) that were reasonably well-defined by most of their items. Although these S-factors remained more weakly defined than the G-factor, this is typical of bifactor models where S-factors reflect only what is uniquely shared by these items once the variance explained by the G-factor has been accounted for (i.e., a form of deviation, or imbalance, from participants’ global levels of FWB; Morin et al., 2020). Indeed, and as noted by others (e.g., Perreira et al., 2018; Morin et al., 2020), reliability estimates are almost systematically lower in a bifactor solution where item-level true score (i.e., reliable) variance is used to define two distinct factors (thus is essentially divided in two), whereas item-level random measurement error is not. For this reason, these authors suggest that the omega coefficient of composite reliability associated with S-factors from a bifactor model should be considered to be satisfactory as long as they remain higher than 0.500. In contrast and tentatively supporting Sorgente and Lanz’s (2019) assertion that this factor could be used to provide a quick summary of participants’ global levels of FWB, the GS items did not seem to retain any specificity beyond their role in the definition of the G-factor (*|λ|* = 0.005–0.286; *M_|λ|_* = 0.123; *ω* = 0.326).

In this bifactor-ESEM solution, cross-loadings also appeared to be substantially lower than in ESEM (*|λ|* = 0.001–0.362; *M_|λ|_* = 0.071). Furthermore, this solution also supported our previous expectations that items HM2, GS1, MM2, and FF5 (as well as many other items) would prove to be stronger indicators of the global factor (respectively, −0.692, 0.807, 0.748, and 0.733) than of their *a priori* specific factors (respectively, 0.024, −0.223, 0.339, 0.305), thus explaining their ESEM cross-loading pattern. However, the problems related to the association of items GS7 and FF4 with the wrong factor (respectively, −0.327 and 0.362) relative to their own factor (respectively, 0.034 and 0.111) remained.

Overall, these results supported the superiority of the bifactor-ESEM solution, while suggesting that two of the items might have been associated with the wrong factors. As a result, we considered an alternative bifactor-ESEM factor structure in which item GS7 was associated with the S-factor HM (rather than GS) and item FF4 was associated with the S-factor MM (rather than FF). It is important to note that, in bifactor-ESEM, each item is allowed to load on all six factors (the G-factor and the five S-factors), meaning that alternative models in which the items are targeted to correspond to other factors, as long as they include the same items and the same number of factors, are equivalent models (Herschberger and Marcoulides, 2013), and will thus always result in the same level of fit to the data and degrees of freedom. The fit of this alternative model is thus identical to that of the previous bifactor-ESEM solution. The same applies to alternative ESEM solutions, but not to alternative CFA and bifactor-CFA solutions. For interested readers, we re-estimated all four models using the new specification of these two items. These additional results are reported in Supplementary Tables S5–S8 of the online supplements and support our conclusions regarding the superiority of the bifactor-ESEM solution. The parameter estimates from the final bifactor-ESEM solution are reported in Table 6 and support our previous conclusions, revealing a strong global factor well-defined by most indicators (|*λ*| = 0.544–0.873, M = 0.727; *ω* = 0.979), accompanied by reasonably well-defined HM (*|λ|* = 0.003–0.546; *M_|λ|_* = 0.339; *ω* = 0.620), PC (*|λ|* = 0.309–0.379; *M_|λ|_* = 0.346; *ω* = 0.554), MM (*|λ|* = 0.325–0.528; *M_|λ|_* = 0.412; *ω* = 0.788), and FF (*|λ|* = 0.291–0.645; *M_|λ|_* = 0.462; *ω* = 0.720) S-factors. The loadings of most items on the G-factor were higher than on their *a priori* S-factor (24 items out of 25), further supporting the strength of the G-factor (Fadda et al., 2020). Conversely, the GS items (*|λ|* = 0.011–0.315; *M_|λ|_* = 0.161; *ω* = 0.388), once again retained only little specificity once their contribution to the G-factor was taken into account, further supporting their theoretical role as direct indicators of participants’ global levels of FWB (Sorgente and Lanz, 2019). In this solution, items GS7 and FF4 now contributed to the definition of the G-factor (respectively, 0.654 and 0.740) and of their new target S-factors (respectively, −0.342 and 0.404). No problematic cross-loading was identified in this solution, which was retained for further analyses.

**Table 6:** Standardized parameter estimates from the final bifactor-ESEM solution for the MSFWBS.

Item (original)	Item (Final)	G-*λ*	HM *λ*	PC *λ*	GS *λ*	MM *λ*	FF *λ*	*δ*
HM1	HM1	**−0.665**	**0.437**	*−0.009*	*0.015*	*0.063*	*−0.018*	0.228
HM2	HM2	**−0.697**	***0.030***	*0.051*	*0.075*	0.190	*0.073*	0.276
HM3	HM3	**−0.670**	**0.546**	*0.014*	*−0.001*	*−0.019*	*0.028*	0.184
GS7	HM4	**0.654**	**−0.342**	*−0.091*	*0.031*	*0.002*	*0.088*	0.438
PC1	PC1	**0.755**	*−0.085*	**−0.379**	*−0.074*	*0.064*	*0.033*	0.268
PC2	PC2	**−0.641**	*0.108*	**0.350**	*−0.001*	*0.057*	*−0.007*	0.385
PC3	PC3	**−0.681**	*−0.098*	**0.309**	0.135	0.188	0.099	0.216
GS1	GS1	**0.815**	*0.030*	*−0.074*	***−0.191***	*−0.052*	*−0.056*	0.288
GS2	GS2	**−0.704**	*0.099*	*0.049*	***−0.041***	*0.050*	*0.078*	0.443
GS3	GS3	**0.802**	*−0.066*	*−0.002*	***0.023***	*−0.014*	*−0.040*	0.350
GS4	GS4	**0.850**	0.118	*−0.015*	***0.055***	*0.029*	*0.034*	0.258
GS5	GS5	**−0.636**	*0.068*	*−0.137*	***−0.315***	*−0.017*	*0.055*	0.405
GS6	GS6	**0.794**	*0.047*	*0.019*	**0.284**	0.169	*0.062*	0.253
GS8	GS7	**0.873**	*−0.007*	*−0.031*	***0.127***	*0.056*	*−0.016*	0.218
GS9	GS8	**0.859**	*0.057*	*0.071*	**0.254**	*0.018*	*−0.009*	0.188
GS10	GS9	**0.853**	*0.013*	*0.022*	***−0.011***	*−0.068*	*−0.016*	0.266
MM1	MM1	**0.725**	*0.070*	*0.098*	*−0.076*	**0.528**	*−0.014*	0.174
MM2	MM2	**0.749**	*0.039*	0.162	*−0.072*	**0.325**	*−0.045*	0.298
MM3	MM3	**0.746**	−0.110	*−0.021*	0.170	**0.348**	0.172	0.251
MM4	MM4	**0.782**	*0.029*	*0.056*	*0.050*	**0.453**	*0.051*	0.174
FF4	MM5	**0.740**	*0.005*	*−0*.142	*0.140*	**0.404**	0.091	0.242
FF1	FF1	**0.651**	*0.041*	*−0.102*	*−0.015*	0.109	**0.400**	0.392
FF2	FF2	**0.544**	*−0.017*	*0.066*	*−0.002*	*0.037*	**0.645**	0.282
FF3	FF3	**0.554**	*−0.051*	*0.106*	*−0.057*	−0.118	**0.513**	0.398
FF5	FF4	**0.724**	*0.001*	*−0.124*	*0.137*	0.311	**0.291**	0.260

*λ*, Standardized factor loading; *δ*, Standardized item uniquenesses; G-, Global factor from a bifactor measurement model; S-, Specific factors from a bifactor measurement model; HM, Having money; PC, Peer Comparison; GS, General subjective financial well-being; MM, Money Management; FF, Financial Future; Item labels are reported in Appendix; Main (target) factor loadings are bolded; Non statistically significant parameters are marked in italics (*p* > 0.05).

### Measurement invariance of scores obtained on the MSFWBS

The results pertaining to the tests of measurement invariance conducted on the final bifactor-ESEM solution are reported in Table 7. These results reveal that all multi-group solutions were associated with an acceptable level of fit to the data. These results also supported the complete invariance of this solution as a function of participants’ age groups (18–29 vs. over 30) and personal income groups (<40 K annually vs. 40 K annually or more), as none of the tests of measurement invariance involving these groups resulted in a decrease in model fit higher than the aforementioned criteria. Similarly, these results supported the configural, weak, strong, and latent means invariance of the solution as a function of participants’ sex (male vs. female) or household income groups (<90 K annually vs. 90 K annually or more). However, strict invariance was rejected for both of these grouping variables. Examination of the parameter estimates from the previous solution of strong invariance and of the modification indices of the failed solution of strict invariance suggested that this lack of strict invariance seemed related to a single item (item MM1 for sex, and GS7 for household income). Relaxing the equality constraints on these uniquenesses resulted in models of partial strict invariance that were supported by the data. These results indicated that item MM1 (“I am satisfied with the way I manage my money”) was slightly more reliable among males (*δ* = 0.102) than females (*δ* = 0.279), and that item GS7 (“I am satisfied with my present financial situation”) was slightly more reliable among participants with an annual household income >90 K (*δ* = 0.151) than those with a household income below 90 K (*δ* = 0.271). Finally, although the invariance of the latent variances and covariances was supported across male and female participants, it was not supported as a function of household income groups. Although tests of partial latent variance covariance invariance cannot be implemented in ESEM (or bifactor-ESEM), the results suggest that this lack of invariance was due to a slightly lower level of variability among participants with an annual household income >90 K (variances = 0.268–0.662; *M* = 0.470) than among those earning <90 K annually (variances fixed to 1 for identification purposes).

**Table 7:** Tests of measurement invariance of the final bifactor-ESEM solution of responses to the MSFWBS.

	*χ*^2^ (df)	CFI	TLI	RMSEA	90% CI	Δ*χ*^2^ (Δdf)	ΔCFI	ΔTLI	ΔRMSEA
*Age (18–29 vs. Over 30)*									
Configural invariance	656.231 (316)^*^	0.950	0.906	0.070	0.062; 0.077				
Weak invariance	689.030 (436)^*^	0.963	0.949	0.051	0.044; 0.058	246.000 (120)	+0.013	0.043	−0.019
Strong invariance	732.351 (454)^*^	0.959	0.946	0.053	0.046; 0.060	46.355 (18)	−0.004	−0.003	+0.002
Strict invariance	782.275 (479)^*^	0.956	0.944	0.053	0.047; 0.060	80.463 (25)^*^	−0.003	−0.002	0.000
Latent variance–covariance invariance	841.122 (501)^*^	0.950	0.940	0.055	0.049; 0.062	58.015 (22)	−0.006	−0.004	+0.002
Latent means invariance	864.168 (508)^*^	0.948	0.938	0.056	0.050; 0.063	63.028 (7)^*^	−0.002	−0.002	+0.001
*Sex (males vs. females)*									
Configural invariance	670.949 (316)^*^	0.949	0.902	0.070	0.063; 0.078				
Weak invariance	752.055 (436)^*^	0.954	0.937	0.057	0.050; 0.063	258.639 (120)	+0.005	+0.035	−0.013
Strong invariance	777.579 (454)^*^	0.953	0.938	0.056	0.049; 0.063	30.102 (18)^*^	−0.001	+0.001	−0.001
Strict invariance	920.369 (479)^*^	0.936	0.920	0.064	0.057; 0.070	67.648 (25)^*^	−0.017	−0.018	+0.008
Partial strict invariance (free uniq. MM1)	784.457 (478)^*^	0.956	0.944	0.053	0.046; 0.060	42.111 (24)	+0.003	+0.006	−0.003
Latent variance–covariance invariance	814.879 (500)^*^	0.954	0.945	0.053	0.046; 0.059	48.939 (22)	−0.002	+0.001	+0.000
Latent means invariance	850.249 (507)^*^	0.950	0.941	0.055	0.048; 0.061	28.988 (7)^*^	−0.004	−0.004	+0.002
*Personal income (<40 K annually vs. 40 K annually or more)*									
Configural invariance	528.982 (316)^*^	0.967	0.938	0.055	0.046; 0.063				
Weak invariance	628.298 (436)^*^	0.970	0.959	0.044	0.036; 0.052	219.481 (120)	+0.003	+0.021	−0.011
Strong invariance	675.983 (454)^*^	0.966	0.955	0.047	0.039; 0.054	41.006 (18)^*^	−0.004	−0.004	+0.003
Strict invariance	757.267 (479)^*^	0.957	0.946	0.051	0.044; 0.057	111.941 (25)^*^	−0.009	−0.009	+0.004
Latent variance–covariance invariance	815.240 (501)^*^	0.952	0.942	0.053	0.046; 0.059	105.489 (22)^*^	−0.005	−0.004	+0.002
Latent means invariance	854.346 (508)^*^	0.947	0.937	0.055	0.049; 0.061	52.327 (29)	−0.005	−0.005	+0.002
*Household income (<90 K annually vs. 90 K annually or more)*									
Configural invariance	508.307 (316)^*^	0.970	0.943	0.052	0.044; 0.060				
Weak invariance	648.349 (436)^*^	0.967	0.955	0.047	0.039; 0.054	245.508 (120)	−0.003	+0.012	−0.005
Strong invariance	664.708 (454)^*^	0.967	0.957	0.045	0.038; 0.053	12.112 (18)	0.000	+0.002	−0.002
Strict invariance	767.280 (479)^*^	0.955	0.944	0.052	0.045; 0.058	147.932 (25)^*^	−0.012	−0.013	+0.007
Partial strict invariance (free uniq. GS7)	720.704 (478)^*^	0.962	0.953	0.048	0.040; 0.054	103.063 (24)^*^	−0.005	−0.004	+0.003
Latent variance–covariance invariance	833.357 (500)^*^	0.948	0.938	0.054	0.048; 0.061	153.31 (22)^*^	−0.014	−0.015	+0.006
Latent means invariance	780.625 (485)^*^	0.954	0.944	0.052	0.045; 0.059	94.868 (8)^*^	−0.008	−0.009	+0.004

*χ*^2^, robust chi-square test of exact fit; df, degrees of freedom; CFI, comparative fit index; TLI, Tucker-Lewis index; RMSEA, root mean square error of approximation; 90% CI, 90% confidence interval; Δ, Change from the previously retained model.

* *p* < 0.01.

### Convergent validity of scores obtained on the MSFWBS

The fit of the model including the final bifactor-ESEM representation of the MSFWBS and all convergent measures was acceptable [*χ*^2^ = 2,008.954; df = 940; CFI = 0.924; TLI = 0.905; RMSEA = 0.050 (90% CI = 0.047–0.053)]. Likewise, the fit of the model including the final bifactor-ESEM representation of the MSFWBS and the complete CFPB was also acceptable [*χ*^2^ = 919.229; df = 431; CFI = 0.946; TLI = 0.926; RMSEA = 0.050 (90% CI = 0.045–0.054)]. Importantly, the decision to rely on a bifactor-ESEM representation of the PFWBS was supported by the comparison of CFA, bifactor-CFA, ESEM, and bifactor-ESEM solutions reported in Supplementary Tables S1, S2 of the online supplements. The final bifactor-ESEM solution revealed a G-factor well-defined by most indicators (*|λ|* = 0.297–0.822; *M_|λ|_* = 0.618; *ω* = 0.911), accompanied by a reasonably well-defined current situation S-factor (*|λ|* = 0.358–0.714; *M_|λ|_* = 0.554; *ω* = 0.778). However, the future expectations items mainly served to define the G-factor, retaining little specificity of their own once their contribution to the G-factor were accounted for (*|λ|* = 0.002–0.473; *M_|λ|_* = 0.229; *ω* = 0.455).

Results from these analyses of convergent validity are reported in Table 8, and reveal strong statistically significant correlations between the MSFWBS G-Factor and the FAS (*r* = −0.814), CFPB (*r* = 0.936–0.942), and the PFWBS G-Factor (*r* = 0.846). The MSFWBS G-Factor was also moderately correlated with the PFWBS S-factor reflecting participants’ levels of dissatisfaction with their current financial situation (*r* = −0.300). Without surprise, the MSFWBS GS S-factor, as well as the PFWBS future expectations S-factor, which were weakly defined in their final bifactor-ESEM solutions, did not share any statistically significant correlations with any of the other factors. Likewise, the MSFWBS PC S-factor, which taps into a facet of FWB unique to this instrument, was not significantly related to any of the convergent measures. In contrast, the MSFWBS HM S-factor was strongly correlated (*r* = −0.647) with the PFWBS current situation S-factors, and weakly correlated with the CFPB (*r* = −0.178 to −0.219). The MSFWBS MM S-factor was moderately correlated with the FAS (*r* = −0.289) and weakly correlated with the CFPB (*r* = 0.111–0.118). Finally, the MSFWBS FF S-factor was weakly correlated with the FAS (*r* = −0.158) and to the PFWBS G-factor (*r* = −0.211).

**Table 8:** Latent variables correlations: analyses of convergent validity.

Scale	G-Factor	HM	PC	GS	MM	FF
FAS	−0.814^**^	0.113	−0.092	0.123	−0.289^**^	0.158^**^
PFWBS (G-Factor)	0.846^**^	0.115	−0.092	−0.086	0.145	0.211^*^
PFWBS (S-Future)	−0.050	−0.069	0.127	0.376	−0.183	−0.126
PFWBS (S-Current)	−0.300^**^	0.647^**^	−0.084	−0.139	−0.001	0.188
CFPB (Reduced version)	0.936^**^	−0.219^**^	−0.010	0.049	0.111^*^	−0.051
CFPB (Complete version)	0.942^**^	−0.178^**^	0.000	0.055	0.118^**^	−0.030

FAS, Financial Anxiety Scale; CFPB, Consumer Financial Protection Bureau; PFWBS, Perceived Financial Well-Being Scale; MSFWBS, Multidimensional Subjective Financial Well-Being Scale; HM, Having money; PC, Peer Comparison; GS, General subjective financial well-being; MM, Money Management; FF, Financial Future.

* *p* ≤ 0.05 and

** *p* ≤ 0.01.

### Criterion-related validity of scores obtained on the MSFWBS

The fit of the model used to test the criterion-related validity of the final bifactor-ESEM representation of the MSFWBS was acceptable [*χ*^2^ = 1,986.716; df = 1,031; CFI = 0.927; TLI = 0.914; RMSEA = 0.045 (90% CI = 0.042–0.048)]. The results from this model are reported in Table 9. These results first indicate that FWB explains a significant portion of the variance in perceived stress (*R*^2^ = 35.20%), psychological distress (*R*^2^ = 22.30%) and life satisfaction (*R*^2^ = 49.20%). Furthermore, scores on the global FWB factor were significantly associated with all three criterion variables, predicting lower levels of perceived stress and psychological distress, and higher levels of satisfaction with life. Unexpectedly, the FF S-factor positively predicted participants’ levels of psychological distress and perceived stress, whereas none of the other S-factors (HM, PC, GS, and MM) predicted any of the criterion variables.

**Table 9:** Criterion-related validity.

Factor	Perceived stress	Psychological distress	Satisfaction with life
	*β* (SE)	*β* (SE)	*β* (SE)
Global factor	−0.485 (0.049)^**^	−0.396 (0.043)^**^	0.684 (0.036)^**^
Having money (specific factor)	0.013 (0.077)	0.024 (0.056)	0.000 (0.077)
Peer comparisons (specific factor)	−0.149 (0.096)	−0.088 (0.066)	0.115 (0.063)
General subjective financial well-being (specific factor)	−0.244 (0.131)	−0.155 (0.133)	−0.001 (0.110)
Money management (specific factor)	−0.094 (0.059)	−0.018 (0.057)	−0.101 (0.057)
Financial future (specific factor)	0.159 (0.053)^**^	0.183 (0.049)^**^	0.002 (0.051)
*R* ^2^	0.352^**^	0.223^**^	0.492^**^

*β*, Standardized regression coefficient; SE, Standard error of the coefficient; *R*^2^, Proportion of explained variance.

^*^p ≤ 0.05.

** *p* ≤ 0.01.

## Discussion

This study sought to improve the conceptual and operational clarity of FWB *via* a comprehensive evaluation of the psychometric properties of the MSFWBS. This instrument was retained based on a thorough examination of the ways FWB had been previously defined, operationalized, and measured in research. This review of the literature allowed us to propose an operational definition of FWB, aligned with the typical definition of general well-being as encompassing hedonic and eudaimonic components (Keyes et al., 2002), while being more specific to one’s financial situation. Moreover, our psychometric investigation of the MSFWBS led us to propose an optimized factor structure (i.e., relying on bifactor ESEM and moving two items to other dimensions) of scores on this instrument that provides a way for scholars to operationalize this integrative definition *via* a variety of global (global FWB) and specific (HM, PC, GS, MM, FF) indicators. Although previous research on this instrument has been limited to emerging adults (Sorgente and Lanz, 2019), our results supported the generalizability of this factor structures to samples of male and female emerging adults and adults over the age of 30 and across different income levels. Lastly, our results also demonstrated the convergent and criterion-related validity of this factor structure, particularly in relation to the global FWB factor. It is our hope that this novel integrative definition and measure will provide an impetus for increased cohesion in FWB research.

### Theoretical contributions

The central contribution of this study involves the clarification and broadening of our understanding of the multidimensional nature of FWB, and in providing support to the ability of the MSFWBS in providing a psychometrically sound measurement of this construct. More precisely, the optimized structure of the MSFWBS established in the present study provides a comprehensive picture of the duality of FWB as a global construct (the G-factor) reflecting the commonalities among a series of distinct dimensions (HM, PC, GS, MM, and FF), most of which also retain some degree of specificity (the S-factors) beyond the assessment of this global construct. Moreover, the convergent validity of participants’ scores on the global FWB factor was clearly established *via* the demonstration of their strong negative correlations with participants’ levels of financial anxiety (FAS), as well as with their scores on the CFPB, PFWBS G-Factor, and PFWBS S-factor.

The GS items did not capture any construct-relevant specificity once their contribution to the assessment of the global FWB factor was accounted for. On the one hand, this observation supports Sorgente and Lanz’s (2019) assertion that the GS items could possibly provide a shorter one-dimensional measure of FWB, as these items all played a strong role in the definition of the global FWB factor without capturing anything else beyond this G-factor. On the other hand, this shorter measure remains imperfect, given that the global FWB factor was also strongly defined by the items associated with the other dimensions (HM, PC, MM, and FF), which also capture additional information beyond this G-factor. Researchers seeking to rely on the GS subscale as a shorter measure of FWB should thus do so with caution, while properly acknowledging that this shorter measure is only able to provide an incomplete one-dimensional picture of a naturally multidimensional construct.

In addition to their strong contribution to the definition of the global FWB factor, the FF items were those who retained the highest levels of specificity beyond this G-factor. These results are consistent with the importance of taking into account one’s future financial expectations when seeking to obtain a comprehensive assessment of FWB (Porter and Garman, 1993; CFPB, 2015; Brüggen et al., 2017; Kempson and Poppe, 2017; Muir et al., 2017; Netemeyer et al., 2018). Unfortunately, the PFWBS future expectations S-factor was too weakly defined in the present study to allow for a proper test of convergent validity for the FF factor. However, this specific dimension was also found to share associations with the FAS, suggesting that one’s financial future might also be a source of financial anxiety, as well as with the PFWBS G-factor, consistent with the strong role played by the items related to one’s future expectations in the definition of this G-factor.

While they also strongly contributed to the definition of the global FWB factor, the HM, PC, and MM items also all retained a meaningful level of specificity beyond their contribution to the definition of the G-factor. Furthermore, the convergent validity of participants’ scores on the MM S-factor was supported *via* the demonstration of statistically significant correlations with the CFPB and the FAS. The latter correlation suggests that money management difficulties may play a role in financial anxiety. Likewise, participants’ scores on the HM S-factor were also found to share a strong association with the conceptually similar PFWBS current situation S-factor (both factors reflect participants’ dissatisfaction with their current financial situation), and a weaker one with the CFPB. However, and in accordance with the unique nature of this component of FWB (which was not covered in our convergent measures), the PC S-factor did not share any significant associations with the convergent measures. Indeed, although many authors had previously highlighted the importance to account for proper money management (Muir et al., 2017; Sorgente and Lanz, 2017, 2019; Netemeyer et al., 2018) and of having money (CFPB, 2015; Brüggen et al., 2017; Kempson and Poppe, 2017; Muir et al., 2017; Sorgente and Lanz, 2017, 2019; Netemeyer et al., 2018) for the assessment of FWB, Sorgente and Lanz (2019) were the only authors to highlight the importance of a peer comparison component. The present results thus support their proposition but highlight the need for a more thorough examination of convergent validity of this component. It should be noted, that the item with the lowest factor loading on the PC S-factor (PC3: “My peers usually have more money available for free time activities than me”) seems to reflect the intended use of money, in this case leisure, rather than one’s global financial situation. This may explain the weaker factor loading associated with this item, and suggests that future research might consider discarding, or replacing, this item.

In addition to supporting the relevance of the five components of FWB identified by Sorgente and Lanz (2019) and the value of bifactor-ESEM for multidimensional measures of well-being (Morin et al., 2016a, 2020), our results also support the generalizability of the psychometric properties of this optimized MSFWBS factor structure. Indeed, our results supported the complete measurement invariance of this factor structure across age groups (18–29 vs. over 30). Thus, although the MSFWBS was originally designed to assess FWB among emerging adults (18–29 years old), our results support its use among older individuals. With few exceptions, the measurement invariance of this factor model was also supported as a function of male and female participants, and of participants from distinct personal, or household, income groups. In fact, the item-level reliability of only two items was found to differ across subgroups of participants, suggesting that care should be used to account for these differences in the context of group comparisons. More precisely, item MM1 (“I am satisfied with the way I manage my money”) was found to be slightly more reliable among males than among females, whereas item GS7 (“I am satisfied with my present financial situation”) was slightly more reliable among participants with a higher household income. Interestingly, this difference did not generalize to comparisons based on personal income, which could perhaps be associated with the slightly lower level of variability in participants’ scores observed among high income households. This lower variability is consistent with the idea that FWB varies less across richer families than it does across poorer families, recalling Leo Tolstoy famous quote “All happy families are alike; each unhappy family is unhappy in its own way.” In any case, researchers interested in comparing FWB as a function of sex or household income should rely on latent variable models, such as those used in this study, to control for this differential item-level reliability, or consider discarding these items.

Lastly, our study sought to document the criterion-related validity of the MSFWBS. Our results showed that FWB, as assessed by this instrument, played a considerable role in the prediction of participants’ levels of perceived stress (*R*^2^ = 35.20%), psychological distress (*R*^2^ = 22.30%) and life satisfaction (*R*^2^ = 49.20%). These findings are consistent with previous studies highlighting the importance of FWB for general functioning and well-being (Shim et al., 2009; Xiao et al., 2009; Zhang and Cao, 2010; Netemeyer et al., 2018; Sorgente and Lanz, 2019; Espinosa and Rudenstine, 2020). In this regard, our results highlighted the primary role of participants’ global levels of FWB (the G-factor) in the prediction of all of these criterion measures (|*β*| = 0.396–0.684). However, they also revealed that most specific components of FWB (i.e., the HM, PC, GS, and MM S-factors) did not contribute to these predictions beyond the role of the FWB G-factor. They also unexpectedly revealed a positive association between participants’ scores on the FF S-factor and their levels of psychological distress and perceived stress. These results thus indicate that specific levels of confidence in one’s financial future higher than one’s global level of FWB (i.e., keeping in mind that the S-factors reflect deviations from the G-factor), tend to be stressful and possibly distressing for participants. More precisely, these results thus suggest a strong desire for a better future that stands in stark contrast with one’s current financial situation seem to be stressful/distressful for participants. Clearly, future research will be needed to assess whether this unexpected association would be replicated and, more importantly, to conduct a more comprehensive assessment of the criterion-related validity of the various S-factors included in the MSFWBS.

### Limitations and directions for future research

The present study includes some noteworthy limitations to consider when interpreting the results. First, our study relied on a convenience sample of participants who volunteered. As a result, selection bias might have led to an over-recruitment of participants who feel challenged in their FWB. Unfortunately, it is not possible to empirically verify this possibility. Likewise, the nature of our sample also makes it hard to generalize our findings to the general population, particularly with respect to language, education, income, as well as the proportion of women. In this regard, it would appear important to verify the extent to which our results would generalize to more diversified samples of participants recruited to be more representative of the general population, as well as from different countries, cultures, and linguistic groups. Second, our study solely relied on self-reported data, suggesting that a variety of self-report biases might have played a role in the results. It is, however, fortunate that common method bias is unlikely to inflate the associations observed in the context of multivariate analyses such as those conducted in this study (Siemsen et al., 2010). In any case, future studies should try to build upon the current investigation through the incorporation of additional sources (e.g., participant’s partner) as well as objective data (e.g., physiological measure of stress). Third, although the present study advances our understanding of FWB and its measurement, it is not yet clear at what point an individual’s level of FWB might be considered sufficient or problematic. Although the score on a FWB measurement scale is certainly not the only predictor of more general components of health and well-being, future studies would do well to identify some threshold of FWB and to recognize that it is significant enough to warrant intervention. Fourth, our study examined the measurement of FWB at a single point in time, without considering its temporal stability. As a result, it would be important for future research to assess how FWB may vary over time, as well as the directionality of its associations with criterion-related measures. Future research should also place more emphasis on FWB outcomes (e.g., quality of life, happiness, compensatory behavior, physical health). Fifth, a deeper understanding of the determinants of FWB could certainly help shed light on the temporal variations of FWB. To this end, Prawitz et al. (2006) noted the presence of 58 potential determinants of FWB, whereas Sorgente and Lanz (2017) noted 95. Among potential determinants and correlates, special attention should be paid to financial literacy, a topic increasingly studied by researchers since the 2008 financial crisis (e.g., Abdullah and Chong, 2014; Goyal and Kumar, 2021). Importantly, financial literacy seems to be more than a simple matter of knowledge (Remund, 2010; Lee et al., 2020; OECD/INFE, 2020; Goyal and Kumar, 2021), making it critical for upcoming research to differentiate between financial knowledge (e.g., Mitchell and Lusardi, 2015) and financial skills and self-efficacy (Warmath and Zimmerman, 2019) in research on financial literacy. Although Remund (2010) argued for a standard way of conceptualizing, operationalizing and measuring financial literacy, such a standard approach is still currently lacking, as noted by Goyal and Kumar’s (2021) systematic review of the relevant research literature. However, research evidence seems relatively clear regarding the role of financial literacy as a determinant of FWB (i.e., Braunstein and Welch, 2002; Santini et al., 2019; OECD/INFE, 2020), highlighting the need for additional research on the associations between different components of financial literacy and FWB. Sixth, items GS7 and FF4 seemed to be initially assigned to the wrong factor and were reassigned to different factors in the present study. Pending replication of the present results, it would thus seem important for future research to conduct a systematic re-assessment of the face validity and content validity of these items. Lastly, unlike Keyes et al. (2002) and other conceptualizations of FWB, the MSFWBS does not separately consider the affective vs. cognitive nature of FWB, simply relying on the incorporation of some affective items in one of its dimensions (GS). The impact of cognition vs. affect for FWB measurement is thus another issue that may be worth considering in future research (e.g., CFPB, 2015; Muir et al., 2017; Netemeyer et al., 2018).

## Conclusion

Our study suggests that, despite their inherent differences, the operational definitions of FWB that have been proposed thus far do indeed reflect a multidimensional construct that can be reliably and validly assessed using the MSFWBS, pending some adjustments to its factor structure. More generally, our study emphasizes the importance of conceptual clarity as well as the reliance on an empirically supported operationalization to foster a greater consensus among FWB researchers. Such a consensus would make it possible for theory and research may move forward in a coherent manner, and thus better serve the interests of researchers, practitioners, and organizations. Although further research is warranted to validate some elements of the MSFWBS, we argue that, at this stage of research, it would be beneficial to prioritize the use of our optimized version of the scale to better guide research on FWB and ensure better comparability among studies.

## Data availability statement

The raw data supporting the conclusions of this article will be made available by the authors, without undue reservation.

## Ethics statement

The studies involving human participants were reviewed and approved by Comité d’éthique de la recherche de l’Université du Québec à Trois-Rivières. The patients/participants provided their written informed consent to participate in this study.

## Author contributions

MA: conception of the study under the supervision of CF, data collection, draft of the manuscript, and analysis and interpretation of the data under the supervision of AJSM. AJSM, CF, and NC: critical review of the manuscript. All authors contributed to the article and approved the submitted version.

## Conflict of interest

The authors declare that the research was conducted in the absence of any commercial or financial relationships that could be construed as a potential conflict of interest.

## Publisher’s note

All claims expressed in this article are solely those of the authors and do not necessarily represent those of their affiliated organizations, or those of the publisher, the editors and the reviewers. Any product that may be evaluated in this article, or claim that may be made by its manufacturer, is not guaranteed or endorsed by the publisher.

## Appendix

Items labels for the multi-dimensional subjective financial well-being scale.

**Table:**

Item # Original	Item # Final	Label
HM1	HM1	*Sometimes I miss funds to buy things I need*
HM2	HM2	*I cannot do some things with my friends, because I do not have the money to do them*
HM3	HM3	*Sometimes I do not have the money to buy what I need*
PC1	PC1	My financial situation is better than my peers’ one
PC2	PC2	*My financial situation is worse than my friends’ one*
PC3	PC3	*My peers have usually more money available for free time activities than me*
GS1	GS1	I have enough money to pursue my passions
GS2	GS2	*I have less money than I need*
GS3	GS3	I cannot complain about my financial situation
GS4	GS4	I am satisfied with how my life is going from a financial point of view
GS5	GS5	*I am constantly stressed because of my financial situation*
GS6	GS6	I am calm about my financial situation
GS7	**HM4**	I have enough funds for everything I need
GS8	GS7	I am satisfied with my present financial situation
GS9	GS8	I am comfortable with my current financial situation
GS10	GS9	I have enough funds to enjoy my life
MM1	MM1	I am satisfied with the way I manage my money
MM2	MM2	I am satisfied with the way I spend my money
MM3	MM3	I feel I can handle my financial situation
MM4	MM4	I am satisfied with the way I manage my financial situation
FF1	FF1	In the near future, I will have enough money to carry my plans out
FF2	FF2	I expect to be very satisfied with the financial situation that I will achieve thanks to my commitment
FF3	FF3	The study/work path I have undertaken will allow me to achieve a satisfying financial situation
FF4	**MM5**	I am satisfied with the way I am preparing myself to reach my long-term goals (for example. to buy a car)
FF5	FF4	I’m on the right track to meet my financial goals

HM, Having money; PC, Peer comparisons; GS, General subjective financial well-being; MM, Money management; FF, Financial future; Italics, Reversed-score items.
