# Supplementary material for: Financial well-being: Capturing an elusive construct with an optimized measure
Source: Front Psychol. 2022 Aug 12;13:935284. doi: 10.3389/fpsyg.2022.935284 (PMC9412911; doi:10.3389/fpsyg.2022.935284)
Supplement: Supplementary file 1 [file Data_Sheet_1.pdf]

Table S1

Fit of the Alternative Measurement Models Estimated for the PFWBS

	χ2	df	CFI	TLI	RMSEA	RMSEA 90% CI
CFA	204.623*	34	.890	.855	.105	.092; .119
Bifactor-CFA	57.725*	25	.979	.962	.054	.036; .072
ESEM	130.248*	26	.933	.884	.094	.078; .110
Bifactor-ESEM	43.443*	18	.984	.959	.056	.035; .077

Note. * p < .01; χ^2: robust chi-square test of exact fit; *df*: degrees of freedom; CFI: comparative fit index; TLI: Tucker-Lewis index; RMSEA: root mean square error of approximation; 90% CI: 90% confidence interval; CFA: Confirmatory factor analysis; ESEM: Exploratory structural equation modeling.

Table S2

Standardized Parameter Estimates of the Alternative Measurement Models Estimated for the PFWBS

	CFA		B-CFA			ESEM			B-ESEM			
	λ	δ	G-λ	S-λ	δ	Future-λ	Current-λ	δ	G-λ	Future-λ	Current-λ	δ
Future1	.633	.599	.681	*.031*	.536	**.511**	-.200	.580	**.658**	***.002***	-.121	.552
Future2	.699	.511	.749	*.084*	.432	**.661**	-*.061*	.513	**.752**	***-.010***	*.015*	.434
Future3	.642	.588	.775	-*.096*	.390	**.607**	-*.058*	.587	**.807**	***-.287***	.100	.257
Future4	.901	.188	.761	.644	.006	**.933**	*.052*	.183	**.822**	**.473**	*.013*	.100
Future5	.889	.209	.754	.401	.271	**.904**	*.020*	.203	**.797**	**.373**	-*.029*	.225
ω	.871			.491		.864				.455		
Current1	.690	.525	-.558	.404	.525	-.241	**.523**	.522	**-.542**	-*.054*	**.419**	.527
Current2	.683	.534	-.602	.338	.524	-.262	**.488**	.545	**-.585**	*.058*	**.358**	.526
Current3	.689	.525	-.291	.731	.382	.222	**.875**	.410	**-.297**	-*.010*	**.714**	.402
Current4	.804	.354	-.556	.561	.375	-*.076*	**.746**	.372	**-.551**	*.045*	**.571**	.368
Current5	.742	.449	-.379	.701	.364	.104	**.839**	.386	**-.373**	-*.072*	**.706**	.357
ω	.845		.907	.775			.844		.911		.778	
Correlation	-.624					-.579						

Note: λ: Standardized factor loadings; δ: Standardized item uniquenesses; G-: Global factor from a bifactor measurement model; S-: Specific factors from a bifactor measurement model; ω: Omega coefficient of composite reliability; CFA: Confirmatory factor analysis; B-CFA: Bifactor-CFA; ESEM: Exploratory Structural Equation Modeling B-ESEM: Bifactor-ESEM; Main ESEM (target) factor loadings are bolded. Non statistically significant parameters are marked in italics (p > .05).

Table S3

Standardized Parameter Estimates from the Measurement Model for the Convergent Measures

Item	PFWBS G-Factor-λ	Future S-λ	Current S-λ	δ	FAS λ	δ	CFPB (Reduced) λ	δ	CFPB (Complete) λ	δ
Item 1	**.675**	***.004***	-.148	.523	.610	.606			.668	.695
Item 2	**.737**	***.026***	*.006*	.457	.748	.431			-.685	.676
Item 3	**.809**	***-.275***	*.082*	.263	.775	.353			-.832	.807
Item 4	**.812**	**.479**	*.026*	.111	.724	.265			-.536	.703
Item 5	**.793**	**.390**	*.010*	.220	.678	.540	.768	.410	.999	.688
Item 6	**-.552**	-*.050*	**.375**	.529	.720	.293	.809	.345	.843	.381
Item 7	**-.589**	*.041*	**.373**	.506	.825	.302	-.581	.621	-.677	.917
Item 8	**-.297**	*.016*	**.615**	.413	.460	.782	-.738	.454	-.873	.661
Item 9	**-.552**	*.038*	**.568**	.345			.695	.517	.840	.713
Item 10	**-.366**	-*.052*	**.578**	.359			-.659	.560	-.843	.974
ω	.911	.467	.745		.896		.861		.894	
Correlations	1	2	3	4	5					
1. PFWBS (G-factor)	**--**									
2. PFWBS (S-Future)	0	--								
3. PFWBS (S-Current)	0	0	**--**							
4. FAS	-.704	-*.010*	.291	--						
5. CFPB	.836	*.026*	-.448	-.825	--					

Note. PFWBS: Perceived financial well-being scale; FAS: Financial Anxiety Scale; CFPB: Consumer Financial Protection Bureau; λ: Standardized factor loadings; δ: Standardized item uniquenesses; G-: Global factor from a bifactor measurement model; S-: Specific factors from a bifactor measurement model; ω: Omega coefficient of composite reliability; Main ESEM (target) factor loadings are bolded. Non statistically significant parameters are marked in italics ($p > .05$).

Table S4

Standardized Parameter Estimates from the Measurement Model for the Criterion Measures

	Perceived Stress		Psychological Distress		Satisfaction with Life	
Item	λ	δ	λ	δ	λ	δ
Item 1	.717	.487	.786	.382	.826	.318
Item 2	.699	.511	.894	.201	.798	.363
Item 3	.741	.451	.597	.643	.929	.138
Item 4	-.310	.582	.825	.319	.839	.295
Item 5	-.451	.434	.819	.329	.720	.482
Item 6	-.559	.353	.752	.434		
Item 7	-.664	.402				
Item 8	.532	.717				
Item 9	-.427	.606				
Item 10	-.685	.413				
Item 11	.572	.673				
Item 12	.354	.875				
Item 13	-.351	.812				
Item 14	.750	.438				
ω	.887		.904		.914	
Correlations	1.	2.	3.			
1. Perceived Stress	--					
2. Psychological Distress	.825	--				
3. Satisfaction with Life	-.502	-.497	--			

Note. λ: Standardized factor loadings; δ: Standardized item uniquenesses; ω: Omega coefficient of composite reliability; All coefficients are statistically significant ($p \leq .05$).

Table S5

Fit of the Alternative Measurement Models Estimated for the MSFWBS

	$\chi 2$	df	CFI	TLI	RMSEA	RMSEA 90% CI
CFA	740.554*	258	.925	.912	.064	.059; .070
Bifactor-CFA	581.006*	243	.947	.935	.055	.050; .061
ESEM	460.046*	178	.956	.926	.059	.052; .066
Bifactor-ESEM	327.082*	158	.974	.950	.049	.041; .056

Note. * p < .01; χ^2: robust chi-square test of exact fit; *df*: degrees of freedom; CFI: comparative fit index; TLI: Tucker-Lewis index; RMSEA: root mean square error of approximation; 90% CI: 90% confidence interval; CFA: Confirmatory factor analysis; ESEM: Exploratory structural equation modeling.

Table S6

Factor Correlations for the CFA and ESEM Measurement Models Estimated for the Revised MSFWBS

	CFA				ESEM			
	HM	PC	GS	MM	HM	PC	GS	MM
PC	-.797**				-.531**			
GS	-.819**	.849**			-.693**	.660**		
MM	-.712**	.741**	.886**		-.499**	.384	.664	
FF	-.704**	.743**	.829**	.848**	-.580**	.456	.654**	.615**

Note. * $p \leq .05$; ** $p \leq .01$; CFA: Confirmatory factor analysis; ESEM: Exploratory structural equation modeling; HM: Having money; PC: Peer Comparison; GS: General subjective financial well-being; MM: Money Management; FF: Financial future.

Table S7

Standardized Parameter Estimates from the Alternative Measurement Models Models Estimated for the Revised MSFWBS

	CFA		B-CFA			ESEM						B-ESEM						
Item	λ	δ	G-λ	S-λ	δ	HM λ	PC λ	GS λ	MM λ	FF λ	δ	G- λ	HM λ	PC λ	GS λ	MM λ	FF λ	δ
HM1	.826	.274	-.648	.481	.222	**.688**	*-.013*	*-.123*	*.041*	*-.049*	.230	**-.665**	**.437**	*-.009*	*.015*	*.063*	*-.018*	.228
HM2	.674	.299	-.660	*.063*	.268	***.139***	*-.275*	*-.475*	.140	*-.025*	.287	**-.697**	***.030***	*.051*	*.075*	.190	*.073*	.276
HM3	.850	.271	-.660	.553	.207	**.854**	*.001*	*.007*	*-.086*	*.053*	.184	**-.670**	**.546**	*.014*	*-.001*	*-.019*	*.028*	.184
GS7	-.741	.450	.656	-.338	.455	**-.546**	*.095*	*.026*	*.050*	.146	.440	**.654**	**-.342**	*-.091*	*.031*	*.002*	.088	.438
PC1	.880	.225	.755	-.543	.135	*-.219*	***.559***	*-.095*	*.231*	*.133*	.285	**.755**	*-.085*	**-.379**	*-.074*	*.064*	*.033*	.268
PC2	-.731	.410	-.633	.306	.406	*.238*	***-.467***	*-.021*	*-.046*	*-.092*	.419	**-.641**	*.108*	**.350**	*-.001*	*.057*	*-.007*	.385
PC3	-.700	.198	-.640	.260	.285	*-.038*	**-.649**	*-.262*	*.066*	*-.023*	.202	**-.681**	*-.098*	**.309**	.135	.188	.099	.216
GS1	.775	.400	.790	*.246*	.315	*-.079*	.381	***.357***	*.080*	*.058*	.334	**.815**	*.030*	*-.074*	***-.191***	*-.052*	*-.056*	.288
GS2	-.694	.470	-.692	*.011*	.450	.229	*-.151*	**-.432**	*-.028*	*.029*	.442	**-.704**	*.099*	*.049*	***-.041***	*.050*	*.078*	.443
GS3	.800	.360	.799	*.048*	.360	-.191	*.127*	**.491**	*.079*	*.034*	.351	**.802**	*-.066*	*-.002*	***.023***	*-.014*	*-.040*	.350
GS4	.852	.274	.852	*.005*	.274	*.082*	*.161*	**.591**	*.135*	*.162*	.257	**.850**	.118	*-.015*	***.055***	*.029*	*.034*	.258
GS5	-.658	.550	-.638	.367	.399	*.149*	*.247*	***-.731***	*-.049*	*.034*	.431	**-.636**	*.068*	*-.137*	***-.315***	*-.017*	*.055*	.405
GS6	.830	.312	.830	-.296	.224	*.006*	*-.079*	***.581***	*.276*	*.144*	.283	**.794**	*.047*	*.019*	**.284**	.169	*.062*	.253
GS8	.890	.209	.883	*-.037*	.218	*-.103*	*.102*	***.566***	*.175*	*.064*	.218	**.873**	*-.007*	*-.031*	***.127***	*.056*	*-.016*	.218
GS9	.872	.240	.866	*-.147*	.228	*.003*	*-.069*	***.810***	*.102*	*.083*	.202	**.859**	*.057*	*.071*	**.254**	*.018*	*-.009*	.188
GS10	.831	.310	.837	*.117*	.286	*-.080*	*.167*	**.584**	*.028*	*.095*	.280	**.853**	*.013*	*.022*	***-.011***	*-.068*	*-.016*	.266
MM1	.859	.262	.733	.498	.214	*.009*	*.051*	*.135*	**.772**	*-.018*	.234	**.725**	*.070*	*.098*	*-.076*	**.528**	*-.014*	.174
MM2	.802	.357	.741	.324	.346	*-.036*	*.012*	*.344*	**.522**	*-.026*	.353	**.749**	*.039*	.162	*-.072*	**.325**	*-.045*	.298
MM3	.844	.288	.781	.287	.308	-.197	*-.048*	*.110*	**.501**	.228	.262	**.746**	*-.110*	*-.021*	.170	**.348**	*.172*	.251
MM4	.914	.165	.799	.460	.150	*-.028*	*.012*	*.197*	**.701**	*.064*	.170	**.782**	*.029*	*.056*	*.050*	**.453**	*.051*	.174
FF4	.849	.278	.779	.312	.297	*-.055*	*.135*	*.058*	**.629**	*.126*	.262	**.740**	*.005*	-.142	*.140*	**.404**	*.091*	.242
FF1	.791	.374	.667	.362	.424	*.042*	.170	*-.016*	*.139*	**.631**	.391	**.651**	*.041*	*-.102*	*-.015*	.109	**.400**	.392
FF2	.703	.506	.546	.691	.225	*.018*	*-.095*	*-.034*	*-.076*	**.957**	.286	**.544**	*-.017*	*.066*	*-.002*	*.037*	**.645**	.282
FF3	.623	.612	.536	.496	.466	*-.058*	*-.065*	*.145*	-.248	**.789**	.427	**.554**	*-.051*	*.106*	*-.057*	-.118	**.513**	.398
FF5	.871	.241	.766	.234	.358	*-.029*	*.086*	*.011*	.446	**.421**	.279	**.724**	*.001*	*-.124*	*.137*	.311	**.291**	.260

Note: λ: Standardized factor loading; δ: Standardized item uniquenesses; G-: Global factor from a bifactor measurement model; S-: Specific factors from a bifactor measurement model; CFA: Confirmatory factor analysis; ESEM: Exploratory structural equation modeling; HM: Having money; PC: Peer Comparison; GS: General subjective financial well-being; MM: Money Management; FF: Financial Future; Item labels are reported in Appendix A; Main ESEM (target) factor loadings are bolded; Non statistically significant parameters are marked in italics ($p > .05$).

Table S8

Composite Reliability (ω) Estimates from the Alternative Measurement Models Models Estimated for the Revised MSFWBS

	CFA	Bifactor-CFA	ESEM	Bifactor-ESEM
G-Factor		.978		.979
HM	.881	.641	.813	.620
PC	.865	.598	.756	.554
GS	.943	.371	.904	.388
MM	.931	.729	.884	.788
FF	.837	.683	.850	.720

Note. CFA: Confirmatory factor analysis; ESEM: Exploratory Structural Equation Modeling; HM: Having money; PC: Peer Comparison; GS: General subjective financial well-being; MM: Money Management; FF: Financial Future; G-factor: Global factor from a bifactor measurement model.
